# Polyol-Made Luminescent and Superparamagnetic β-NaY_0.8_Eu_0.2_F_4_@γ-Fe_2_O_3_ Core-Satellites Nanoparticles for Dual Magnetic Resonance and Optical Imaging

**DOI:** 10.3390/nano10020393

**Published:** 2020-02-23

**Authors:** Walid Mnasri, Lotfi Ben Tahar, Patricia Beaunier, Darine Abi Haidar, Michel Boissière, Olivier Sandre, Souad Ammar

**Affiliations:** 1 Lab. ITODYS, Université de Paris, CNRS UMR-7086, 75205 Paris, France; walid.tevez@hotmail.fr; 2 Lab. CHO-MN, Faculté des Sciences de Bizerte, Université de Carthage, LR18 ES117021 Zarzouna, Tunisia; bentaharlotfi@gmail.com; 3 Lab. ERRMECe, CY Cergy Paris Université, Maison Internationale de la Recherche, 95031 Neuville-Oise, France; michel.boissiere@u-cergy.fr; 4 Lab. LCPO, Univ. Bordeaux, Bordeaux INP, ENSCPB, CNRS UMR-5629, 33607 Pessac, France; olivier.sandre@enscbp.fr; 5 Faculty of Science of Arar, Northern Border University, 91431 Arar, Saudi Arabia; 6 Lab. LRS, Sorbonne Université, CNRS UMR-7197, 75005 Paris, France; patricia.beaunier@sorbonne-universite.fr; 7 Lab. IJCLab, Université Paris-Saclay, CNRS/IN2P3 UMR-9012, 91405 Orsay, France; abihaidar@imnc.in2p3.fr; 8 Lab. IJCLab, Université de Paris, 91405 Orsay, France

**Keywords:** dual superparamagnetic and luminescent nanoprobes, europium-doped fluoride nanocrystals, iron oxide nanoparticles, polyol process, human foreskin fibroblast cell viability

## Abstract

Red luminescent and superparamagnetic β-NaY_0.8_Eu_0.2_F_4_@γ-Fe_2_O_3_ nanoparticles, made of a 70 nm-sized β-NaY_0.8_Eu_0.2_F_4_ single crystal core decorated by a 10 nm-thick polycrystalline and discontinuous γ-Fe_2_O_3_ shell, have been synthesized by the polyol process. Functionalized with citrate ligands they show a good colloidal stability in water making them valuable for dual magnetic resonance and optical imaging or image-guided therapy. They exhibit a relatively high transverse relaxivity r_2_ = 42.3 mM^−1^·s^−1^ in water at 37 °C, for an applied static magnetic field of 1.41 T, close to the field of 1.5 T applied in clinics, as they exhibit a red emission by two-photon excited fluorescence microscopy. Finally, when brought into contact with healthy human foreskin fibroblast cells (BJH), for doses as high as 50 µg·mL^−1^ and incubation time as long as 72 h, they do not show evidence of any accurate cytotoxicity, highlighting their biomedical applicative potential.

## 1. Introduction

Current biomedical imaging techniques are vital for the diagnosis of various diseases. Each imaging mode has its own merits and disadvantages and uses specific probes with particular physical and chemical properties. As a consequence, a single technique does not encompass all the functionalities required for comprehensive imaging. Therefore, multimodal methods, with enhanced signal sensitivity, better spatial resolution, and ability to relay information about biological systems, at the molecular and cellular levels, are becoming strongly recommended. To achieve such a purpose, it is necessary to design and produce multimodal probes which combine in a single object all these requirements.

For instance, magnetic nanoparticles (NPs) can be used alone as magnetic resonance imaging (MRI) contrast agents, highlighting, when administrated to a patient, abnormal versus healthy tissues [1,2,3]. At the same time, they can serve as platforms on which functional moieties like fluorescent tags [4], biolabels [5,6] or radioactive isotopes [7,8] can be added. By contrast, each of these functional moieties might be used as a substrate implemented by magnetic species. In this context, MRI-optical dual probes consisting of a fluorescent dye-doped silica core surrounded by superparamagnetic iron oxide NPs have been prepared and successfully used to both detect cancer cells and collect subcellular information on them, while applying simultaneously or consequently MRI and fluorescence imaging [9]. Quantum dots (QDs) functionalized on their surface by paramagnetic gadolinium chelates have been also engineered and successfully evaluated for dual MRI-optical in vitro imaging [10]. Up-conversion and down-conversion luminescent lanthanide fluoride nanocrystals (UCLFs and DCLFs) doped with paramagnetic gadolinium cations have been also prepared and successfully tested for image-guided oncological surgery, since MRI allowed the pre-operating tumor localization and local light emission better delimitated the tumor during surgery ablation [11]. This list is not at all comprehensive and other combinations between magnetic centers and optically active species are possible. Focusing on such bimodality, it must be pointed out that materials processing remains a key issue for the production of this kind of two-in-one nano-objects. It must be achieved in judicious operating conditions in order to build nanoconstructs with optimal relaxometric and luminescent properties and minimal side-effects. For this purpose, probes based on magnetic centers attached at the surface of UCLFs or DCLFs are certainly the most valuable to address all these goals. Indeed, thanks to their chemical nature and crystallographic structure, UCLFs and DCLFs usually exhibit stability against photobleaching, photoblinking and photochemical degradation, as they express reduced toxicity risks [12,13,14,15]. But whereas several UCLFs and DCLFs-based paramagnetic-fluorescent architectures were largely developed and described in the literature, seldom are the superparamagnetic-fluorescent ones. Indeed, examples in which UCLFs or DCLFs are decorated at their surface by paramagnetic Gd^3+^ chelates [16,17,18,19] or just doped in their outer crystallographic lattice layer by paramagnetic Gd^3+^ cations are numerous [20,21,22,23], but those combining UCLF or DCLF to superparamagnetic iron oxide nanocrystals are scarce, and most of them are based on an iron oxide core coated by a lanthanide fluoride shell [24,25,26] or a UCLF or a DCLF core coated by very small iron oxide satellites [27]. To fill this gap, we propose in the present work to build a superparamagnetic-luminescent architecture based on these two components within the last geometry, using a single material processing route, the polyol process. The polyol process is a versatile, easy-to-achieve and scalable wet chemistry route, which has already demonstrated its power for the synthesis of well-crystallized and shape-controlled granular hetero-nanostructures [28,29,30]. In practice, europium-doped β-NaYF_4_ single crystals were precipitated in polyol and subsequently dispersed in a fresh iron salt polyol solution to serve as seeds for iron oxide nanocrystal growth, leading to the formation of β-NaYF_4_:Eu@γ-Fe_2_O_3_ core-satellite particles. Their magnetic and optical properties were then investigated with a special emphasis on their ability to be used as MR and optical imaging probes. Therefore, water proton relaxometry, fluorescence spectroscopy and microscopy and in vitro healthy human cell viability assays were specifically performed. All the obtained results are discussed hereafter.

## 2. Experimental Section

### 2.1. Synthesis of Water-Soluble β-NaY_0.8_Eu_0.2_F_4_@γ-Fe_2_O_3_ Nanoprobes

**Figure 1:**
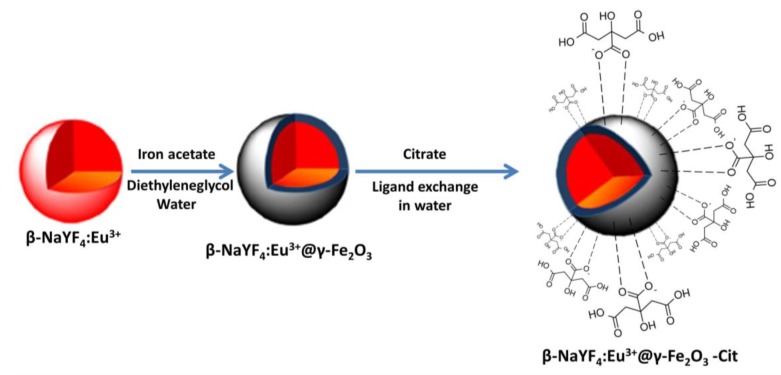
Schematic representation of the chemical strategy used to produce water-dispersible β-NaY_0.8_Eu_0.2_F_4_@γ-Fe_2_O_3_ nanoparticles (NPs). To be as synthetic as possible, only the coordination of citrate ligands through their carboxylate groups to the particle surface was illustrated.

The synthesis of β-NaYF_4_:Eu@γ-Fe_2_O_3_ particles was performed in a three steps route (Figure 1). First, β-NaYF_4_:Eu seeds, with an atomic Eu content of 20%, were produced in polyol. Second, γ-Fe_2_O_3_ nanocrystals were grown around them in a fresh polyol medium. Third, the resulting nanocomposites were functionalized by hydrophilic citrate ligands, making them water-dispersible.

In practice, β-NaY_0.8_Eu_0.2_F_4_ nanocrystals were synthesized in polyol [31]. Appropriate amounts of NaOH (98%, Sigma-Aldrich, St. Quentin-Fallavier, France), NH_4_F (98%, Sigma-Aldrich), Y(CH_3_CO_2_)_3_ (99.9%, Sigma-Aldrich) and Eu(CH_3_CO_2_)_3_ (99.9%, Sigma-Aldrich,) were dissolved in a mixture of 125 mL of ethyleneglycol (≥99%, Sigma-Aldrich) and 62 mL of oleic acid (Fisher scientific, 70%) and heated under reflux for 30 min (6 °C/min). After cooling down to room temperature, the formed precipitate was recovered, by adding an excess of ethanol, through several cycles of centrifugation and distilled water washing. It was then dried in air at 80 °C for a couple of hours.

β-NaY_0.8_Eu_0.2_F_4_@γ-Fe_2_O_3_ NPs were prepared in polyol too by dispersing 300 mg of the as-obtained β-NaY_0.8_Eu_0.2_F_4_ powder in 31 mL of diethyleneglycol (99%, Across Organics,), in which 3 mmol of Fe(CH_3_CO_2_)_2_ (95%, Sigma-Aldrich) and 0.125 mL of deionized H_2_O were added. The resulting mixture was then heated up to reflux for 3 h. The obtained precipitate was then separated from the supernatant, at room temperature, by centrifugation and washing with ethanol and water, and finally dried in air at 80 °C. Freely dispersed γ-Fe_2_O_3_ NPs were also prepared within the same operating conditions, removing the β-NaY_0.8_Eu_0.2_F_4_ seeds from the starting reaction solution.

Citrate grafting was achieved through a simple ligand exchange method, replacing the residual organic moieties at the surface of the fluoride and/or oxide particles by the freshly introduced multivalent citrate species, taking advantage from the complexing ability of their carboxylate and hydroxyl groups [32]. Typically, 1 g of β-NaY_0.8_Eu_0.2_F_4_@γ-Fe_2_O_3_ nanoparticles were dispersed in 200 mL of an aqueous Na_3_[(HO)C(CH_2_CO_2_)_3_] (50 mM) solution. The resulting suspension was mechanically stirred and heated up to 100 °C for 30 min. The resulting precipitate was collected using a strong magnet, then washed with an excess of ethanol, to remove the non-grafted organic species, and finally dried in air at 60 °C.

### 2.2. Structural and Microstructural Characterization

Powder X-ray diffraction (PXRD) was performed on all the produced powders using a X’pertPro diffractometer (Panalytical, Almelo, Netherlands), equipped with a Co-Kα tube (40 kV, 40 mA) and configured for a θ-θ Bragg-Brentano reflection geometry. Highscore Plus software (Panalytical, Almelo, Netherlands) was used for phase identification and peak indexation and MAUD software (version 2.55, Trento, Italy), based on Rietveld refinement [33], was employed for cell parameter and average crystal size determination. X-ray fluorescence spectroscopy (XRF) was also carried out with a Minipal4 spectrometer (Panalytical, Almelo, Netherlands), equipped with a Rh-Kα tube (30 kV, 87 μA). Quantification was achieved via pre-plotted calibration curves using Na^+^, Y^3+^, Eu^3+^ and Fe^3+^ standard solutions.

Transmission electron microscopy (TEM) was conducted on a JEM 2010 UHR microscope (JEOL, Tokyo, Japan), operating at 200 kV and micrographs were collected thanks to a Gatan Orius SC1000 4008 × 2672 pixel charge-coupled device CCD camera (AMETEK, Berwyn, PA, USA). Dynamic light scattering (DLS) experiments were also performed in order to estimate the hydrodynamic size distribution of the produced citrate functionalized nanoparticles. They were carried out in water thanks to a Zetasizer NanoZS instrument (Malvern Panalytical, Worcestershine, UK) equipped with a 5.0 mW He-Ne laser operating at 632.8 nm and detecting scattered light at 90° using an avalanche photodiode detector (APD). 

To complete these analyses, Fourier transformed infrared (FTIR) spectroscopy was performed at ambient temperature using an Equinox FTIR spectrometer (Bruker, Baltimore, MD, USA) operating in a transmission scheme (KBr pellet). Thermogravimetry (TG) analysis was also carried out by heating a given mass of pre- and post-functionalized particles in air up to 1000 °C (5 °C/min) thanks to a SETARAM TGA92 apparatus.

### 2.3. Magnetometry and Relaxometry Measurements

The variation of the magnetization *M* as a function of temperature *T* and as a function of magnetic field *H* was measured on solid state, using a MPMS-5S SQUID magnetometer (Quantum Design, San Diego, CA, US). In practice, 20 mg of the dried powder were slightly compacted in a plastic sampling tube to avoid their movement during measurements. Magnetization was recorded *versus* temperature *M*(*T*), under a dc magnetic field of 200 Oe, operating within zero field cooling (ZFC) and field cooling (FC) conditions, between 5 and 330 K. Also, magnetization was recorded versus magnetic field *M*(*H*) at 310 K (37 °C) by cycling the magnetic field between 70 and −70 kOe.

Relaxometry was carried out on the colloidal state fixing the concentration of particles to 3 g·L^−1^. In practice, 0.7 mL of each solution was introduced in a nuclear magnetic resonance (NMR) tube (7.5 mm outer diameter). The tubes were then inserted in a mq60 relaxometer (Bruker, Baltimore, MD, USA) equipped with a 60 MHz/1.41 T magnet. The *T_1_* and *T_2_* relaxation times were recorded as a function of iron concentrations [Fe], at 310 K. [Fe] was properly calculated by an accurate titration of iron content for all the samples, averaging the values inferred from XRF and colorimetry [34] without any acid mineralization. *T_2_* was measured with a Carr-Purcell-Meiboom-Gill (CPMG) sequence using an inter-echo time (TE) between 0.2 and 4 ms (typically *T_2_*/50) and a mono-exponential decay fit of 150 data points. The recycling delay (RD) was adjusted around 5 times the initial *T_1_* value, measured by an inversion recovery (IR) sequence, whose relaxation was fitted by a mono-exponential on 20 data points, the first delay (TE) being around *T_1_*/10 and the final duration around 3 times *T_1_*. These parameters (TE, RD and amplifier gain) were adjusted until the *T_2_* and *T_1_* values were measured with low uncertainty, typically 0.1%, for each iron concentration (four values in total).

### 2.4. Photoluminescence Measurements

The luminescence properties were measured at room temperature on the prepared colloids (2 mg·mL^−1^). A FluoroMax-4 spectrofluorometer (Horiba Jobin Ivon, Glasgow, UK), working with a 150-W Xe-arc lamp, was used. Excitation wavelength was selected at 396 nm by the grating monochromator. Such a light source usually provides high and continuous excitation intensity.

### 2.5. Cell Culture and in Cellulo Cytotoxicity Assay

Cytotoxicity assays were performed on human foreskin fibroblasts (BJH) cells. These cells are abundant and easy to manipulate for various biomedical applications [35]. They are often reported to evaluate the cytotoxicity of nanomaterials [31,36,37,38,39,40,41], making them useful for the present study. In practice, 48-well cell culture clusters (Corning, NY, USA) were plated with 1 mL of 15,000 cells/mL cell suspension. After 24 h, the cells were treated with various concentrations of particles (10, 25 and 50 μg·mL^−1^) prepared in Dulbecco’s modified Eagle medium (DMEM) supplemented with 10% foetal bovine serum (FBS). Incubation was performed at 37 °C in a 5% CO_2_ humidified incubator for 24, 48 and 72 h. Replicate wells were used for each control and test concentration per plate.

Alamar Blue (AB) assays were performed to test the viability of incubated cells. In practice, the cells were rinsed once with PBS, and 1 mL of AB medium (10% *v*/*v* solution of AB in DMEM) was added to each well. After 3 h incubation, the AB absorbance of the samples was measured at 570 nm (A570) and 600 nm (A600) on a microplate reader and compared to those measured in the absence of NPs. All toxicity experiments were conducted in at least triplicate (three independent experiments). Raw data from cytotoxicity assays were collated and analyzed using Microsoft Excel^®^ (Microsoft Corporation, Redmond, WA, USA). Cytotoxicity was expressed as the mean percentage inhibition relative to the unexposed control ± standard deviation (SD). Statistical analyses from cytotoxicity assays were carried out using one-way analyses of variance (ANOVA) followed by Dunnett’s multiple comparison tests. Statistical significance was accepted at *p* ≤ 0.05 for all tests.

### 2.6. Confocal and Two-Photon Microscopies

Optical microscopy was performed to visualize the morphology of the cells before and after NPs incubation. In practice BJH cells were seeded on glass coverslips 24 h before treatment with the same NP doses than previously for a unique incubation time of 48 h. Fixed cells (4% paraformaldehyde) were mounted with mounting medium (Vector Laboratories, Burlingame, CA). Their nuclei and their cytoskeleton were counterstained with (4′,6-diamidino-2-phenylindole) abbreviated as DAPI (λ_ex_ = 405 nm) and 7-[(*4R*)-5-[[[[4-[3,6-Bis(dimethylamino)xanthylium-9-yl] -3-carboxyphenyl]amino]thioxomethyl]amino]-4-hydroxy-L-leucine]phalloidin abbreviated as TRITC (λ_ex_ = 630 nm). Within these operating conditions, NPs cannot be detected by their own emitted red light, due to its superposition with that of labelled cytoskeleton. To detect them, the same slides were observed in parallel with a two-photon microscope, a LEICA TCS SP8 MP FLIM system (PIMPA platform, Paris Saclay University, Orsay, France). The excitation source of this microscope is a femtosecond pulsed infrared laser (Ti: Sapphire). Two hybrid detectors (Leica, Germany), in non-descanted position were used to optimize the detection of the NPs’ red fluorescence. The spectral analysis was achieved in the confocal scanning head of the microscope which pilots the grating and mirror in front of it. The spectral resolution was 10 nm, covering the range from 380 nm to 780 nm.

## 3. Results and Discussion

### 3.1. Structural and Microstructural Properties

**Figure 2:**
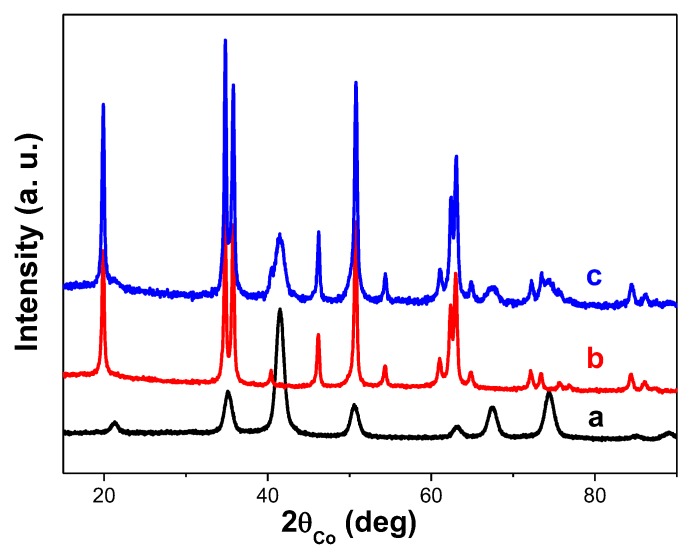
Powder X-ray diffraction (PXRD) patterns of (**a**) γ-Fe_2_O_3_, (**b**) β-NaY_0.8_Eu_0.2_F_4_ and (**c**) β-NaY_0.8_Eu_0.2_F_4_@γ-Fe_2_O_3_ NPs.

**Figure 3:**
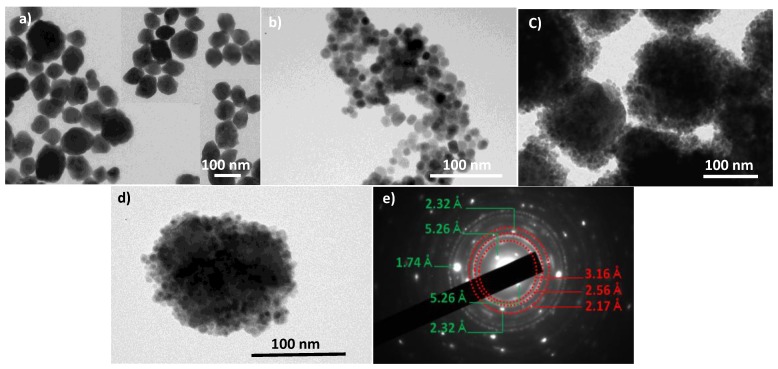
TEM images of (**a**) β-NaY_0.8_Eu_0.2_F_4_, (**b**) γ-Fe_2_O_3_ and (**c**) β-NaY_0.8_Eu_0.2_F_4_@γ-Fe_2_O_3_ NPs. (**d**) Micrograph of one representative composite particle and (**e**) its selected electron diffraction pattern (SAED). Note that the pattern can be fully indexed within the β-NaYF_4_ hexagonal structure (light spots and related distances in green) and the γ-Fe_2_O_3_ cubic one (dashed lines and related distances in red).

The structure and the microstructure of the as-produced composite particles were first checked by PXRD and TEM. The recorded PXRD pattern and the collected TEM micrographs are given in Figure 2 and Figure 3, respectively. The same analyses were performed on β-NaY_0.8_Eu_0.2_F_4_ and γ-Fe_2_O_3_ NPs prepared separately. 

The PXRD pattern of the composite particles matched very well the superposition of those of β-NaYF_4_ (ICDD No. 98-005-1917) and γ-Fe_2_O_3_ (ICDD No. 98-008-7119). The cell parameter values were refined and found to be close to those tabulated for bulk materials and close to those previously reported on β-NaY_0.8_Eu_0.2_F_4_ [31] and γ-Fe_2_O_3_ [42] nanocrystals prepared by soft chemistry (Table 1). The Rietveld fit quality was illustrated in Figure S1 in the supporting information section, through the perfect superposition of the experimental and calculated patterns. 

The average crystallographic coherent domains size <L_XRD_> as well as the refined average micro-strain-induced lattice deformation <ε> were also refined for each crystalline phase and the values obtained were summarized in Table 1, agreeing fairly with the production of almost strain free nanocrystals. The crystal size of the oxide phase prepared alone or in presence of the fluoride particles, is about 9 nm, and that of the fluoride phase prepared alone or decorated by the oxide particles is about 70 nm. These values are very close to the average diameters observed for each phase by TEM (Figure 3), suggesting that both fluoride and oxide phases, prepared alone or together, are consistent with single crystals. The core-forming NPs are quite polygonal in shape with an average length of 70 nm, while the shell-forming ones look like spheres with an average diameter of 9 nm.

Interestingly, the iron oxide shell around the europium doped yttrium fluoride core appeared as constituted by the aggregation of several maghemite single crystals. Compared to the previous works on UCLF or DCLF core coated by iron oxide satellites [27], the iron oxide shell of our engineered multimodal probes is also discontinuous but thicker and denser, suggesting a higher magnetization in the resulting composite NPs, which is important for MRI modality. Note also that the final size of our probes, core and shell together, is consistent with an average value smaller than 100 nm, which is crucial for *in vivo* applications, in term of in-body diffusion after intravenous (IV) administration.

**Figure 4:**
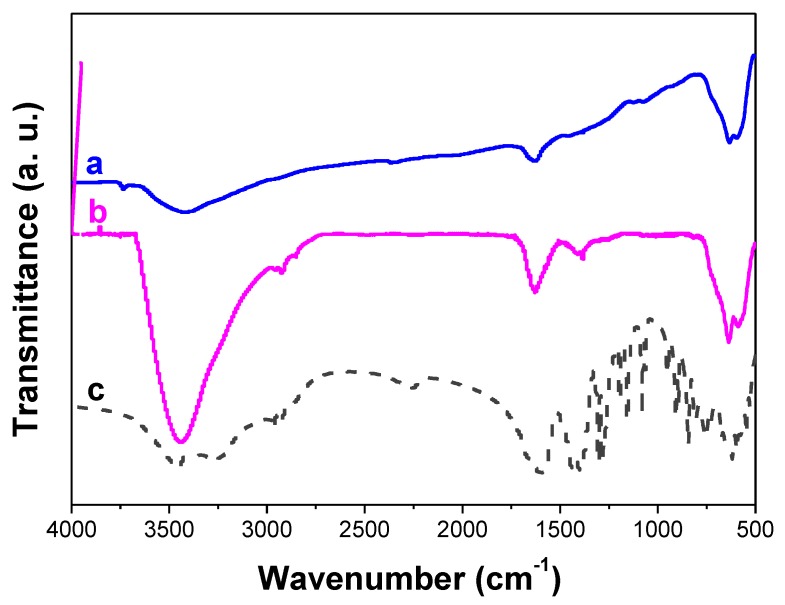
Fourier transformed infrared (FTIR) spectra of the (**a**) as-produced β-NaY_0.8_Eu_0.2_F_4_@γ-Fe_2_O_3_ particles and (**b**) their related citrated coated counterparts, compared to (**c**) that of sodium citrate salt.

The recorded electron diffraction pattern on a representative composite particle is consistent with the superposition of a Scherrer-like pattern, fully indexed in the spinel γ-Fe_2_O_3_ structure, and a Laue-like one, corresponding to the β-NaYF_4_ structure, confirming the polycrystalline arrangement of the shell and the single crystalline nature of the core. The 3.16, 2.56 and 2.17 Å distances measured on the main diffraction rings are consistent with the (022), (311), (004) spinel crystallographic planes, respectively. Those measured on the main spots, namely 5.26, 2.32 and 1.74 Å, correspond to the (010), (111) and (002) hexagonal fluoride crystallographic planes, respectively (Figure 3). TEM observations were also carried out on citrate-coated composite particles (not shown) and, as expected, they highlighted the ability of adsorbed citrate ligands to reduce particle aggregation, since isolated and well-defined core-shell particles can be distinguished in the recorded micrographs. This feature was confirmed by DLS measurements. Indeed, a monomodal size distribution was recorded for the composite colloid with an average hydrodynamic diameter of about 130 nm (Figure S2). The discrepancy between this value and that inferred from TEM observations, suggests that a 20–30 nm-thick organic layer (mainly citrates), including hydration water molecules, surrounds the core-shell inorganic particles. This hydrophilic outer layer is particularly useful for water diffusion close to the engineered magnetic probes during future MRI experiments. Indeed, such a hydrophilic layer would contribute to improving the relaxing properties of the magnetic iron oxide nanocrystals, at the surface of the fluoride core, toward the nuclear magnetic moments of diffusing water protons. Citrate grafting was confirmed by FTIR spectroscopy on dried particles, by comparing the spectrum recorded on these particles to those collected on non-functionalized particles and on sodium citrate salt, respectively (Figure 4). 

The characteristic symmetric (1390 cm^−1^) and antisymmetric (1590 cm^−1^) stretching bands of the carboxylate C=O groups of the citrate entities were clearly evidenced in the spectrum after citrate functionalization. Also, the symmetric and antisymmetric stretching bands of alkyl C–H groups of the citrate ions were located at 2880 and 2930 cm^−1^, respectively. The FTIR bands ascribed to these organic groups are strong indications of the grafting of citrates on to the particle’s surface. In addition, inorganic features are also clearly evidenced on both the FTIR spectra of pre- and post-functionalized particles by the appearance of two broad intense bands at low frequencies at 637 and 587 cm^−1^, respectively. They are attributed to β-NaY_0.8_Eu_0.2_F_4_ [31] and γ-Fe_3_O_3_ [42]_,_ respectively.

Finally, TG analysis allowed us to quantify the outer organic content (mainly citrates) on the functionalized composite particles (Figure S3). A value of about 30 wt.% was determined, which is quite high, revealing a significant grafting density and a quite complete coverage by a hydrophilic layer of the engineered probes.

### 3.2. Magnetic Properties

**Figure 5:**
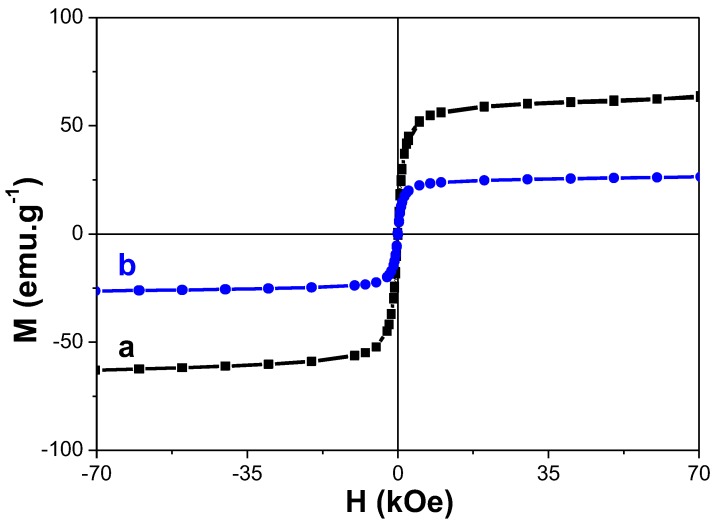
Variation of the magnetization as function of the magnetic field *M*(*H*) measured at *T*=310 K of (**a**) γ-Fe_2_O_3_ and (**b**) β-NaY_0.8_Eu_0.2_F_4_@γ-Fe_2_O_3_ particles.

β-NaY_0.8_Eu_0.2_F_4_ core-forming composite particles are mainly diamagnetic. The paramagnetic Eu^3+^ contribution is too small and only the addition of γ-Fe_2_O_3_ nanocrystals to their surface may give a non-zero magnetization under an applied magnetic field to the final hetero-nanostructure. Therefore, focusing on the intrinsic magnetic properties of these particles, we measured their isothermal hysteresis loop in their powder-state, without citrate coating, and we compared it to that of free γ-Fe_2_O_3_, at 310 K, namely 37 °C, the physiological temperature (Figure 5). For the two systems, a typical superparamagnetic behavior was evidenced. The curve of magnetization as a function of the applied magnetic field is completely reversible, without any coercivity nor remanence. It is clearly seen that the specific magnetization of bare maghemite powder decreases significantly when these nanocrystals are decorating β-NaY_0.8_Eu_0.2_F_4_ cores. The saturation magnetization of each system was determined (per gram of powder). Designating the saturation magnetisation of individual β-NaY_0.8_Eu_0.2_F_4_ (assumed to be zero), bare γ-Fe_2_O_3_ NPs and their related β-NaY_0.8_Eu_0.2_F_4_@γ-Fe_2_O_3_ composites as *M*_sat_(core), *M*_sat_(shell) and *M*_sat_(core-shell), respectively, the maghemite weight content, *x*, in the β-NaY_0.8_Eu_0.2_F_4_@γ-Fe_2_O_3_ core-shell NPs can be deduced from:

*x*·*M*_sat_(shell) + (1 − *x*)·*M*_sat_(core) = *M*_sat_(core-shell) (1)

**Figure 6:**
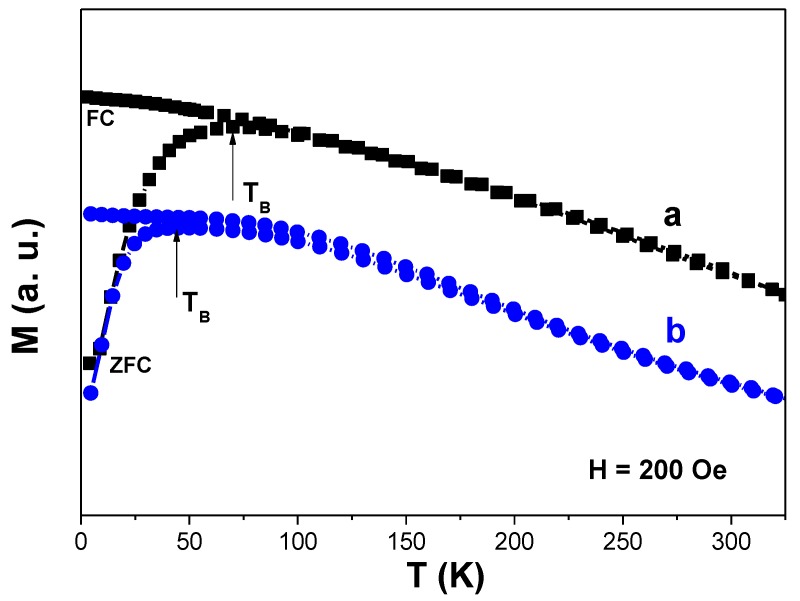
Zero field cooling (ZFC-) and field cooling (FC)-*M*(*T*) curves recorded on the (**a**) as-produced γ-Fe_2_O_3_ and (**b**) β-NaY_0.8_Eu_0.2_F_4_@γ-Fe_2_O_3_ particles.

In the present case, *M*_sat_(shell), the saturation magnetization of pure γ-Fe_2_O_3,_ was found to be equal to 63 emu·g^−1^, and *M*_sat_ (core-shell), the saturation magnetization of β-NaY_0.8_Eu_0.2_F_4_@γ-Fe_2_O_3_, was found to be equal to 25 emu·g^−1^. An iron oxide weight content of about 40 wt.% was thus estimated. This magnetic weight fraction is comparable to the value of 49 wt.% calculated from the fluoride core diameter (67 nm) and the iron shell thickness (9 nm), taking into account their mass densities of 2.5 g·cm^−3^ and 5.2 g·cm^−3^, respectively. It is important to note that although the saturation magnetization value of the composite particles is decreased compared to individual maghemite nanoparticles, it is still strong enough for MRI contrast agent application. The superparamagnetic behavior of these particles was also evidenced by ZFC and FC thermal variation of their magnetization (Figure 6). A net irreversibility between the FC-*M*(*T*) and ZFC-*M*(*T*) branches was observed at low temperature. The average blocking temperature, *T*_B_, which usually characterizes the transition between the blocked ferromagnetic state of the particles and their superparamagnetic one, was measured at the maximum of the ZFC-*M*(*T*) curves. It was found to be about 70 K for pure γ-Fe_2_O_3_ powders and 43 K for β-NaY_0.8_Eu_0.2_F_4_@γ-Fe_2_O_3_ ones. This decrease of the *T*_B_ value usually traduces a reduction of the strength of dipolar interactions between γ-Fe_2_O_3_ magnetic single domains_._ The special arrangement change of these magnetic domains between the two samples may explain that. In the core-shell geometry, these single domains interact strongly between themselves within a same shell but weakly from one shell to another.

The same magnetic measurements were performed on dried citrate–coated β-NaY_0.8_Eu_0.2_F_4_@γ-Fe_2_O_3_ nanoparticles (not shown) and the same superparamagnetic behavior was observed with some small differences. At first, a saturation magnetization of 22 emu·g^−1^, smaller than that measured on the non-functionalized particles, due to the diamagnetic organic contribution of the citrate species onto their surface. This decrease is roughly proportional to the citrate weight content estimated by TG (Figure S3), and remains weak compared to the initial value. Moreover, the blocking temperature of the resulting nanohybrids decreases a little bit, the surface ligands contributing to increase the inter-particle distance in the core-shell particles, thus reducing the mutual dipolar interactions between the iron oxide nanocrystals involved.

**Figure 7:**
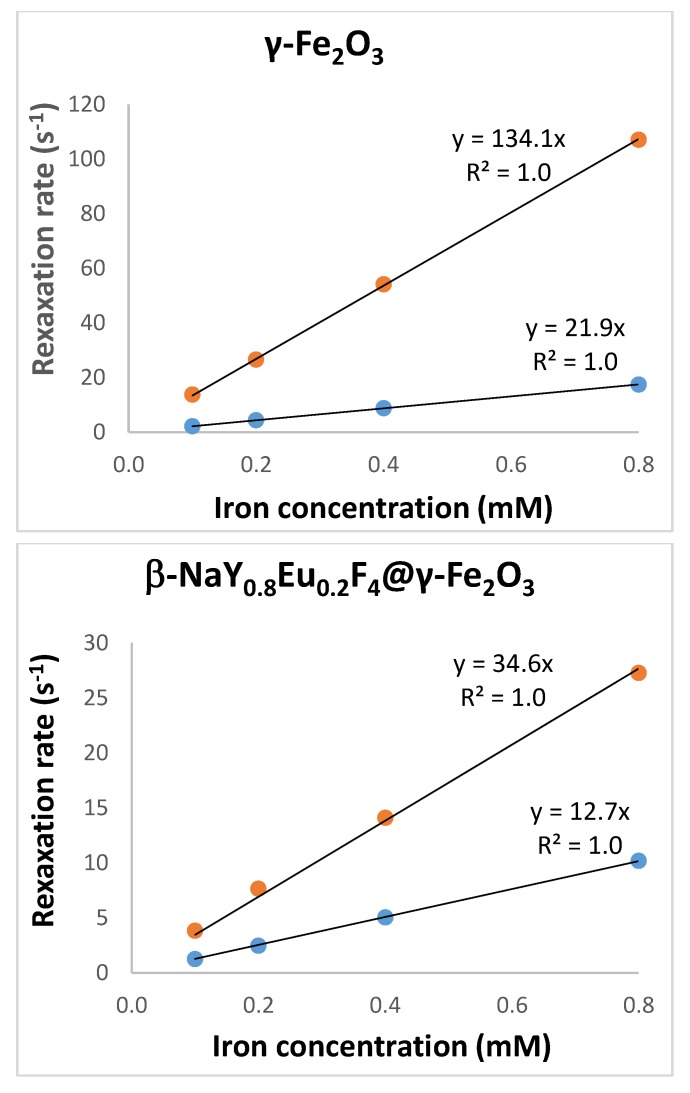
Determination of the longitudinal (*r*_1_) and transverse (*r*_2_) relaxivities of citrate coated samples dispersed in water at 37 °C for an applied static field of 1.41 T, by plotting the relaxation rates (inverse of the relaxation times) for *T*_1_ (blue circles) and *T*_2_ (red circles). The relaxation rates (1/*T*_i = 1 or 2_)_citrate_ of pure citrate solutions were subtracted so that the plots have zero intercept values.

Finally, proton relaxometry measurements were performed on both the citrated γ-Fe_2_O_3_ and the citrated β-NaY_0.8_Eu_0.2_F_4_@γ-Fe_2_O_3_ particles dispersed in water to evaluate their efficiency to relax nuclear spins of water proton with the aim of using them as MRI contrast agents. Their transverse (*r*_2_) and longitudinal (*r*_1_) relaxivities were measured at physiological temperature, 37 °C, with a 60 MHz relaxometer based on a 1.41 Tesla magnet (i.e., close to the 1.5 Tesla magnetic field of most clinical MRI machines used in hospitals). Practically, the longitudinal (*T*_1_) and transverse (*T*_2_) relaxation times of water protons were measured within appropriate spin-echo sequences of radiofrequency pulses, respectively IR and CPMG sequences. The *T*_1_ and *T*_2_ values were measured in pure citrate buffer and for a series of equivalent [Fe] concentration, typically 0.8, 0.4, 0.2 and 0.1 mM. In a second time, these concentrations were double-checked by titration of the stock suspensions by two different analytical methods, namely colorimetry (ultraviolet-visible (UV-Vis) absorbance) and X-ray fluorescence (XRF). Relaxivities were obtained from the slope of the linear variation with [Fe] of the longitudinal (respectively transverse) decay rate of water proton spins, according to:

1/*T*_i = 1 or 2_ = *r_i_*_= 1 or 2_. [Fe] + (1/*T_i_*_= 1 or 2_)_citrate_ (2)

where the relaxation times of a pure citrate solution (*T*_1_ = 3813 ms and *T*_2_ = 650 ms as measured experimentally) are taken into account. The curves obtained are plotted in Figure 7, and the measured relaxivity values are summarized in Table 2.

According to Table 2, we noticed a significant drop concerning the *r*_2_ value of γ-Fe_2_O_3_ from 134.1 to 34.6 s^−1^·mM^−1^ in the case of the core-shell structure due to the decrease of its saturation magnetization, not compensated by the increase clustering effect, *r*_2_ being expected to vary with the square of both the magnetization and the hydrodynamic diameter within the limits of the “outer sphere” theory of superparamagnetic contrast agents [43]. On the other hand, the longitudinal relaxivity *r*_1_ of the core-shells only slightly decreased compared to γ-Fe_2_O_3_ from 21.9 to 12.7 s^−1^·mM^−1^, showing a good access of the water molecules to the iron oxide surface of the shell. Nevertheless, β-NaY_0.8_Eu_0.2_F_4_@γ-Fe_2_O_3_ nanoparticles still remain good candidates as MRI contrast agents, either negative or positive ones, owing to their relatively low *r*_2_/*r*_1_ ratio of around 2.7 [44].

### 3.3. Optical Properties

**Figure 8:**
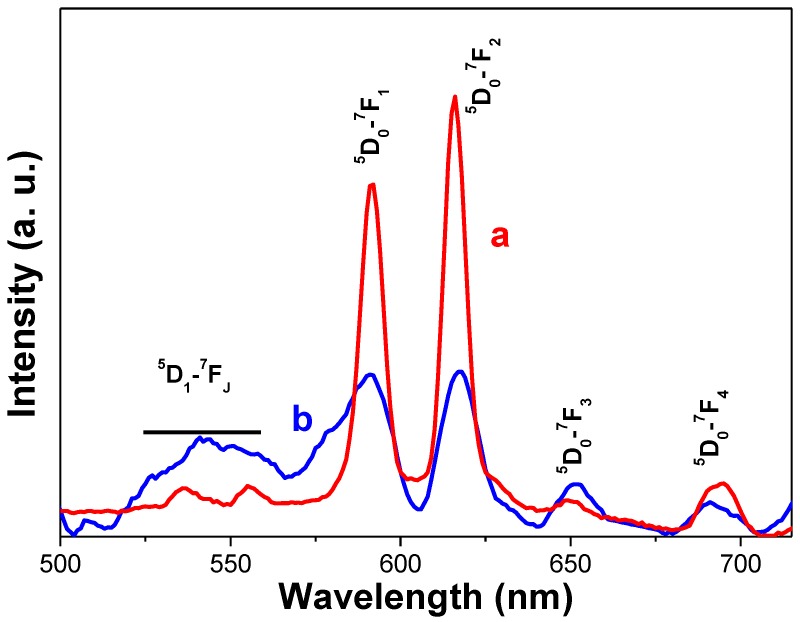
Photoluminescence (PL) spectra of (a) the core and (b) composite based colloids for a continuous excitation at 396 nm.

The optical properties of β-NaY_0.8_Eu_0.2_F_4_ and β-NaY_0.8_Eu_0.2_F_4_@γ-Fe_2_O_3_ particles were measured, in particular the photoluminescence (PL) spectra of their aqueous colloidal suspensions, obtained by dispersing their citrated counterparts in deionized water. Under a continuous excitation at 396 nm, which corresponds to the spin-forbidden ^7^F_0_–^5^L_6_ absorption transition of Eu^3+^ cations [45,46], several sharp emission lines were recorded (Figure 8). They are associated to the radiative relaxation of Eu^3+^ cations from their ^5^D_J_ = 0 electronic state to the ^7^F_J_ =1, 2, 3 and 4 ones, the intensity of the line at 616 nm being the highest. Interestingly, by decorating the β-NaY_0.8_Eu_0.2_F_4_ with a layer of γ-Fe_2_O_3_ crystals, the whole PL core intensity decreased (Figure 8) but remained detectable.

### 3.4. In Cellulo Assays

**Figure 9:**
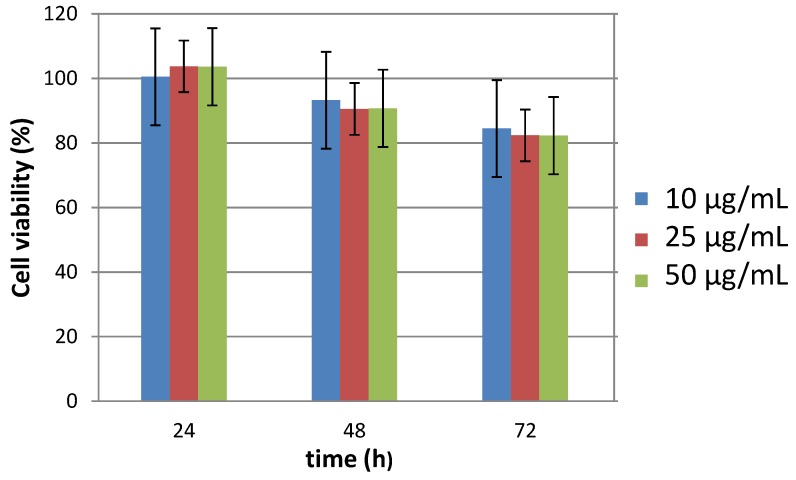
Relative number of viable BJH cells, determined using the Alamar blue test when exposed to different doses of citrated β-NaY_0.8_Eu_0.2_F_4_@γ-Fe_2_O_3_ particles for different contact times. The results were expressed as a ratio to non-stimulated serum-free cultured cells for dose point. (Analysis of variance (ANOVA) test *p* ≤ 0.05).

**Figure 10:**
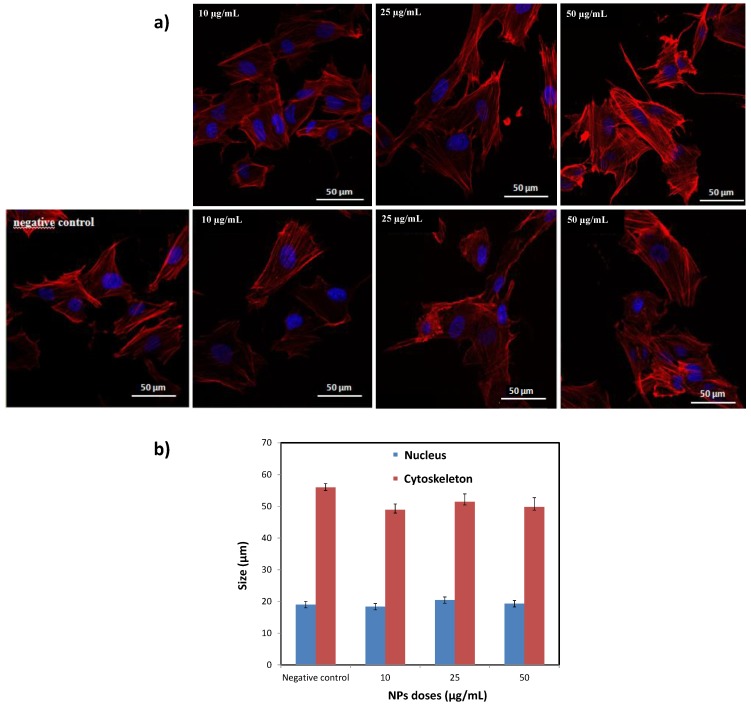
(**a**) Confocal fluorescence microscopy images collected on BJH cells incubated during 48 h with different doses of citrated β-NaY_0.8_Eu_0.2_F_4_@γ-Fe_2_O_3_ particles. Note the nucleus and the cytoskeleton were counterstained with the blue and red emitting DAPI and TRITC dyes, respectively. (**b**) Nucleus and cytoskeleton size distributions (based on the size analysis of a total of 25 cells) were added to appreciate eventual morphological changes after contact with NPs. In the present case there is nothing significant.

To complete our investigations, the viability of BJH human cells incubated by our engineered citrated composite particles was evaluated. Interestingly, these particles appeared to be non-cytotoxic for doses as high as 50 µg·mL^−1^ (Figure 9), without any dose effect. Of course, the cell viability decreased slightly when the time of contact between the particles and the cells increased (up to 72 h) but without inducing a significant detrimental effect. These results were confirmed by cell morphology imaging (Figure 10a). The collected immunofluorescence pictures did not evidence significant changes in the general shape either in nuclei shape or size (Figure 10b).

**Figure 11:**
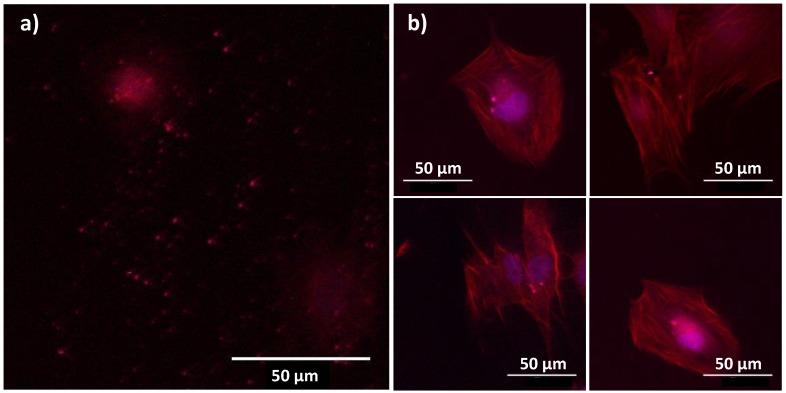
Panel of two photon images collected on BJH cells contacted with citrated β-NaY_0.8_Eu_0.2_F_4_@γ-Fe_2_O_3_ particles (48 h and 50 µg·mL^−1^) and counterstained with blue DAPI and red TRITC dyes. An excitation wavelength of 830 nm was used for this purpose and the images were selected to highlight (**a**) non-internalized particles and (**b**) internalized ones. The particles appear as bright red dots.

Finally, the ability of the produced particles to be used as biomarkers was established by using two-photon imaging microscope), focusing on the citrated β-NaY_0.8_Eu_0.2_F_4_@γ-Fe_2_O_3_ particles in contact with BJH cells (Figure 11). Within the operating conditions (see experimental section) the cell structure can be visualized without hindering the optical signature of the particles. 

Clearly, all the recorded bio-physicochemical properties on these engineered β-NaY_0.8_Eu_0.2_F_4_@γ-Fe_2_O_3_ nanoparticles are very promising. They allow us to highlight the advantages offered by these all-inorganic bimodal MR and optical imaging probes compared to others, combining also superparamagnetism and lanthanide-based luminescence. The list of such probes is short in the relevant literature. To the best of our knowledge, in comparison to present system, equivalent MRI relaxivity values were measured on iron oxide nanocrystals doped by lanthanide ions, Eu^3+^ (typically *r*_1_ = 15.4 and *r*_2_ = 33.9 mM^−1^·s^−1^ in water at 0.47 T and 37 °C), but less optimized optical responses were reported on them. Indeed, the red emission of these nanocrystals was obtained only under a UV excitation of 254 nm [47]. Such high light excitation energy means a high UV-induced cell damage risk, which is a severe limitation for any in cellulo and in vivo use. Replacing spinel oxide by β-NaYF_4_ fluoride allows for decreasing this energy, since less non-radiative de-excitation phenomena may proceed. Quite equivalent MRI relaxivity values were also measured on these alternative probes, based on magnetite cores surrounded by a thin lanthanide doped NaYF_4_ shell (typically Yb^3+^-Er^3+^ [24] or Yb^3+^-Tm^3+^ [26]). However, their optical properties were not optimized, despite the replacement of the matrix of the lanthanide centers. The poor crystalline quality of the fluoride shell (amorphization, in relation with its in-solution growth-processing conditions, contributed to this degradation. Finally, the observed luminescence properties here on our engineered nanoprobes are qualitatively closer to those obtained on an almost similar core-shell structure, in which about 100 nm-sized lanthanide-doped β-NaYF_4_ single crystals were cross-linked to less than 10 nm sized Fe_3_O_4_ particles [27]. But, once again, in relation to their non-optimized material processing conditions, these composite particles exhibit a very weak total magnetization (less than 9 emu·g^−1^ at body temperature, far below the 25 emu·g^−1^ measured here on our nanoprobes), making them less valuable for negative MRI contrasting than ours.

## 4. Conclusions

We have synthesized core-satellite structured β-NaY_0.8_Eu_0.2_F_4_@γ-Fe_2_O_3_ nanoparticles of less than 100 nm in size by the so-called polyol process. These composite particles integrate optical and magnetic dual functions in one single nanoprobe geometry. The γ-Fe_2_O_3_ satellites make the built hetero-nanostructures suitable for *T*_2_-weighted or *T*_1_-weighted MRI, while the β-NaY_0.8_Eu_0.2_F_4_ cores offer the ability to be used as biomarkers. The toxicity of the particles as well as their ability to be used as in vitro biomarkers were tested on healthy BHJ human model cells after citrate functionalization to make them hydrophilic. Interestingly, no cell death was reported from AB viability assays for doses as high as 50 µg·mL^−1^ and incubation time as prolonged as 72 h. All of these interesting results provide a proof of concept that our engineered citrated composite particles are clearly valuable for dual magnetic resonance and optical medical imaging.

## Figures and Tables

**Table 1:** Cell parameters, average crystal size and average micro-strain-induced lattice deformation, as inferred from Rietveld refinement. The average particle size inferred from transmission electron microscope (TEM) observation is given for information.

Samples	Fluoride Phase			Oxide Phase			<*D*_TEM_> (nm)
	*a,c* (Å)	<*L*_XRD_> (nm)	<*ε*>	*a* (Å)	<*L*_XRD_> (nm)	<ε>	
β-NaY_0.8_Eu_0.2_F_4_	5.993(5) 3.530(5)	66	10 × 10^−4^	-	-	-	67(7)
γ-Fe_2_O_3_	-	-	-	8.375(7)	9	18 × 10^−4^	9(2)
β-NaY_0.8_Eu_0.2_F_4_@γ-Fe_2_O_3_	5.992(5) 3.526(5)	72	22 × 10^−4^	8.378(7)	8	22 × 10^−4^	89(9)

**Table 2:** Iron concentration [Fe] of the stock suspensions, as inferred from X-ray fluorescence (XRF) and colorimetry analyses. Average values of these data were taken to calculate the *r*_1_ and *r*_2_ relaxivities and assess the efficiency of the obtained engineered multifunctional probes as magnetic resonance imaging (MRI) contrast agents compared to pure Fe_2_O_3_. Average hydrodynamic diameter and polydispersity index (PDI) as provided by dynamic light scattering (DLS) at 90° scattering angle.

Samples	[Fe] *^a^* mMm	[Fe] *^b^* mM	*r*_1_ s^−1^mM^−1^	*r*_2_ s^−1^mM^−1^	<*D*_DLS_> *^c^* nm	PDI *^c^*
γ-Fe_2_O_3_	23.1	19.0	21.9	134.1	66	0.28
β-NaY_0.8_Eu_0.2_F_4_ @ γ-Fe_2_O_3_	6.6	7.7	12.7	34.6	150	0.20

^a^ measured by colorimetry; ^b^ measured by X-ray fluorescence; ^c^ measured by dynamic light scattering (DLS).

## Supplementary Materials

The following are available online at https://www.mdpi.com/2079-4991/10/2/393/s1. Figure S1: Experimental (scatter) and calculated (red line) XRD patterns of (a) β-NaY0.8Eu0.2F4 and (b) β-NaY0.8Eu0.2F4@γ-Fe2O3 powders. The residue, defined as the difference between the experimental and calculated diffractograms, is given for each sample (blue line) to illustrate the fit quality. The Bragg reliability factor R_B_ ranges around 2 for all the performed refinements. Figure S2: Intensity and number counted hydrodynamic size distribution of citrate coated β-NaY_0.8_Eu_0.2_F_4_@γ-Fe_2_O_3_ composite particles dispersed in pure water. Figure S3: Thermogram of the as-produced β-NaY_0.8_Eu_0.2_F_4_@γ-Fe_2_O_3_ particles and those of their citrated counterparts.
