# Supplementary material for: Polyol-Made Luminescent and Superparamagnetic β-NaY0.8Eu0.2F4@γ-Fe2O3 Core-Satellites Nanoparticles for Dual Magnetic Resonance and Optical Imaging
Source: Nanomaterials (Basel). 2020 Feb 23;10(2):393. doi: 10.3390/nano10020393 (PMC7075321; doi:10.3390/nano10020393)
Supplement: Supplementary file 1 [file nanomaterials-10-00393-s001.pdf]

Supporting Information for:

Polyol-made luminescent and superparamagnetic β-$NaY_{0.8}Eu_{0.2}F_4$@γ-Fe_2O_3 core-satellites nanoparticles for dual magnetic resonance and optical imaging

1. XRD analysis

The recorded XRD patterns on all the produced powders were analysed by the Rietveld method using MAUD software. A pseudo-Voigt function was used for modelling the peak profile. A set of about 20 parameters were then refined, including the scale factor, the zero shift, the background polynomial coefficients, the peak profile parameters, and for each constituting phase, its cell parameter, average crystallographic coherence length (assuming isotropic crystals), average micro-deformation, weight ratio and the isotropic temperature factors for each of its atoms. The atomic coordinates of all atoms of all the involved phases were fixed. To converge rapidly the fitting process, the occupation site ratios were first fixed in respect to the nominal chemical composition for each phase. The great atomic number proximity between iron and cobalt atoms does not allow an accurate estimation of their distribution along the spinel sites nor the rock-salt ones. The starting structural model converged satisfactorily with a R_B reliability factor and a χ^2 coefficient very close to 2% and 1, respectively. The quality of the fit is illustrated through the superposition of the experimental and calculated patterns for both β-$NaY_{0.8}Eu_{0.2}F_4$ and β-$NaY_{0.8}Eu_{0.2}F_4$@γ-Fe_2O_3 powders (Figure S1).

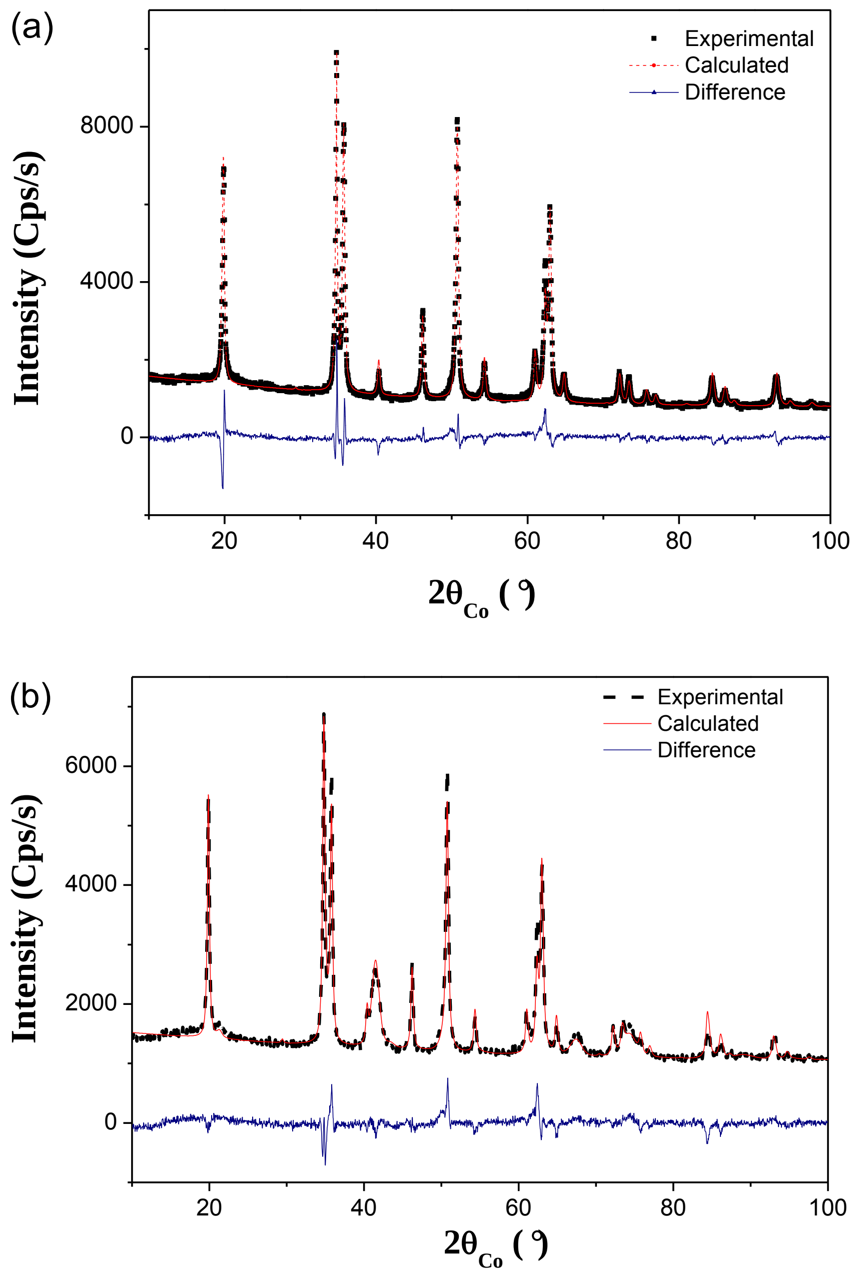

Figure S1. Experimental (scatter) and calculated (red line) XRD patterns of **(a)** β-NaY0.8Eu0.2F4 and **(b)** β-NaY0.8Eu0.2F4@γ-Fe2O3 powders. The residue, defined as the difference between the experimental and calculated diffractograms, is given for each sample (blue line) to illustrate the fit quality. The Bragg reliability factor R_B ranges around 2 for all the performed refinements.

2. DLS analysis

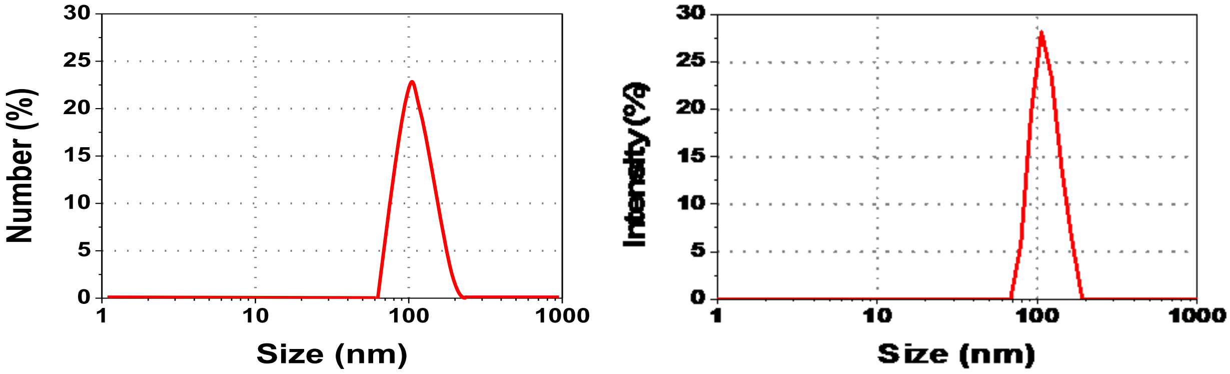

Figure S2. Intensity and number counted hydrodynamic size distribution of citrate coated β-$NaY_{0.8}Eu_{0.2}F_4$@γ-Fe_2O_3 composite particles dispersed in pure water.

3. TG analysis

The thermogram of citrated and non-citrated β-NaY0.8Eu0.2F4@γ-Fe2O3 particles were recorded in air. Typically, the former exhibits two main departures, that of physisorbed water (before 100°C) and that of chemisorbed residual polyol (between 200 and 300°C). The latter exhibited a more important organic departure between 300 and 500°C, attributed to citrate decomposition.

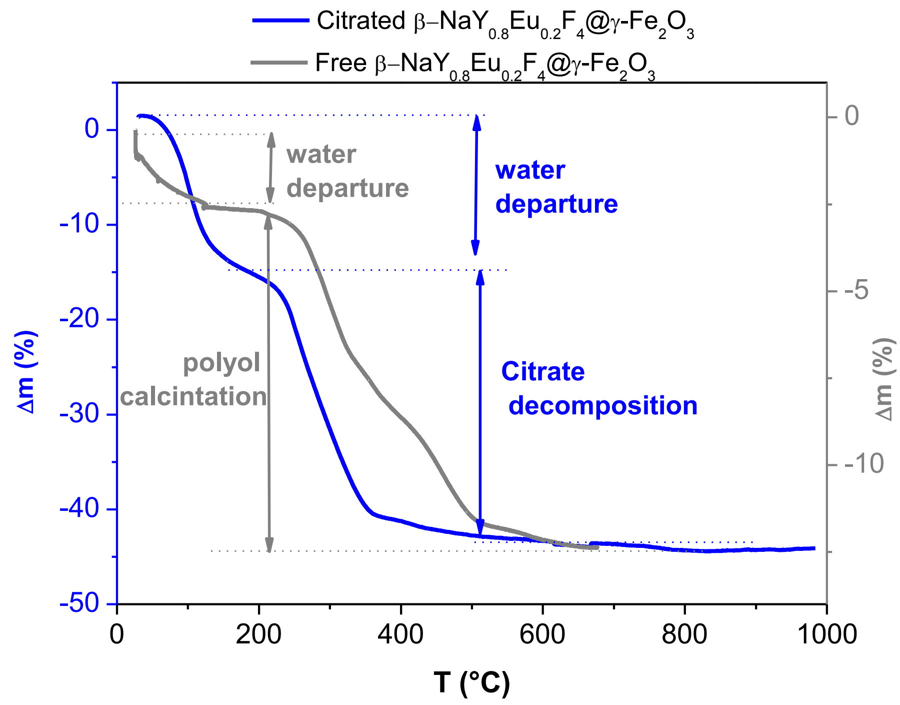

Figure S3. Thermogram of the as-produced β-$NaY_{0.8}Eu_{0.2}F_4$@γ-Fe_2O_3 particles and those of their citrated counterparts.
